# Supplementary material for: Synthesis of bimetallic MOFs via interface control using gallium-based liquid metal
Source: Nat Commun. 2025 Nov 28;16:11721. doi: 10.1038/s41467-025-66942-y (PMC12753776; doi:10.1038/s41467-025-66942-y)
Supplement: Supplementary file 1 — Supplementary Information [file 41467_2025_66942_MOESM1_ESM.pdf]

Supporting Information

Synthesis of Bimetallic MOFs via Interface Control Using Gallium-Based Liquid Metal

*Jui-Chi Lin[1], Chun-Tse Wei[1], Chien-Hua Wang[2],Yu-Cheng Chang[1] ,Wen-Wei Wu[2] and Chun-Wei Huang[1]**

[1] *Department of Materials Science and Engineering, Feng Chia University, Taichung 407102, Taiwan*

[2] *Department of Materials Science and Engineering, National Yang Ming Chiao Tung University, Hsinchu 30010, Taiwan*

*Correspondence and requests for materials should be addressed to C-W. H.

(email: huangcw@fcu.edu.tw)

List of contents

Supplementary Fig. 1 | Electrochemical behavior of Ga and Mg-Ga alloy electrodes.

Supplementary Fig. 2 | Ion release stability under different applied voltages.

Supplementary Fig. 3 | Effect of alloy composition and voltage on the crystallinity of ZnMg-MOF-74.

Supplementary Fig. 4 | Effect of ligand concentration on the crystallinity of ZnMg-MOF-74.

Supplementary Fig. 5 | Time-dependent crystallization of ZnMg-MOF-74.

Supplementary Fig. 6 | Amorphous morphology of ZnMg-MOF-74 after direct annealing in air at 500 ℃.

Supplementary Fig. 7 | Schematic illustration of the two-step annealing process converting ZnMg-MOF-74 into ZnMgO derivatives.

Supplementary Fig. 8 | TEM-EDS elemental mapping of ZnMgO derived from ZnMg-MOF-74 under different two-step annealing conditions.

Supplementary Table 1 | Summary of synthesis conditions and applications of bimetallic MOFs.

Supplementary Table 2 | Average grain area of ZnMgO under different pretreatment temperatures.

Supplementary Table 3 | Photodetection performance of ZnMgO materials, including responsivity, external quantum efficiency, and detectivity.

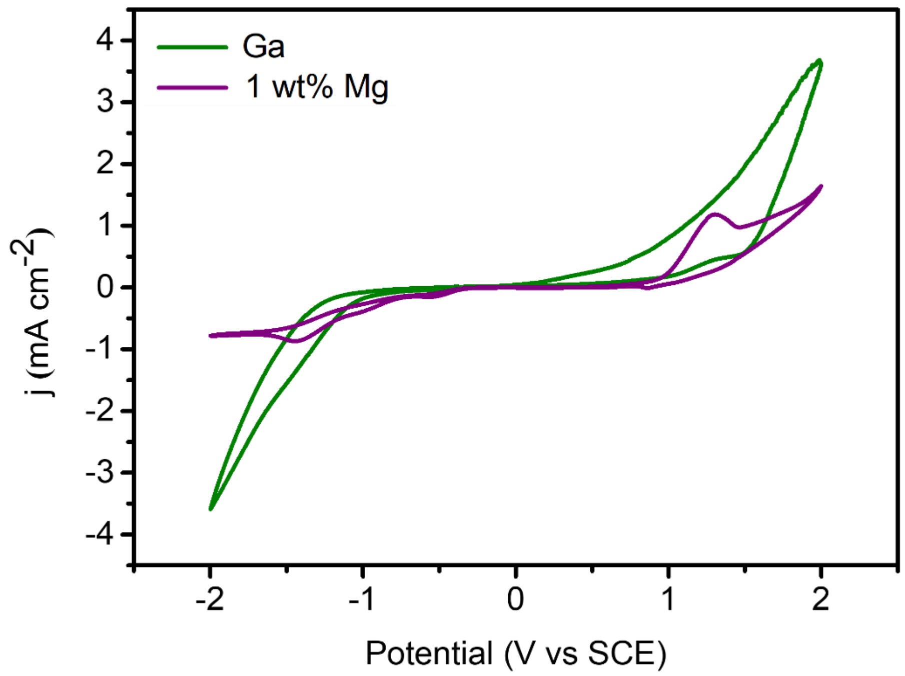

Supplementary Fig. 1 | Electrochemical behavior of Ga and Mg-Ga alloy electrodes.
Cyclic voltammetry reveals Mg oxidation in the alloy, while Ga remains electrochemically inactive.

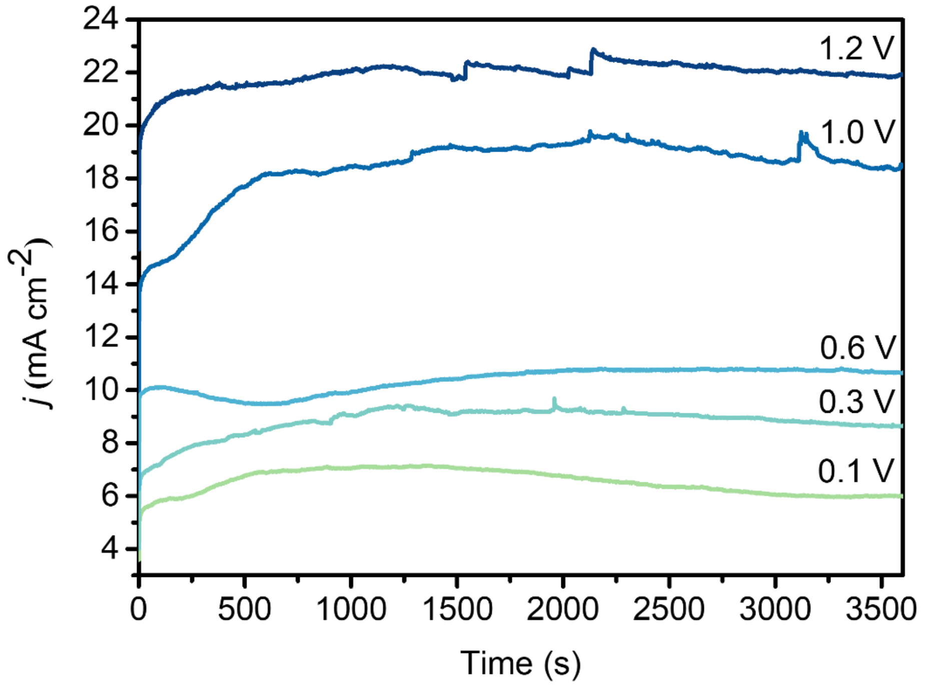

Supplementary Fig. 2 | Ion release stability under different applied voltages.
Time-current curves during ZnMg-MOF-74 synthesis demonstrate stable ion release behavior.

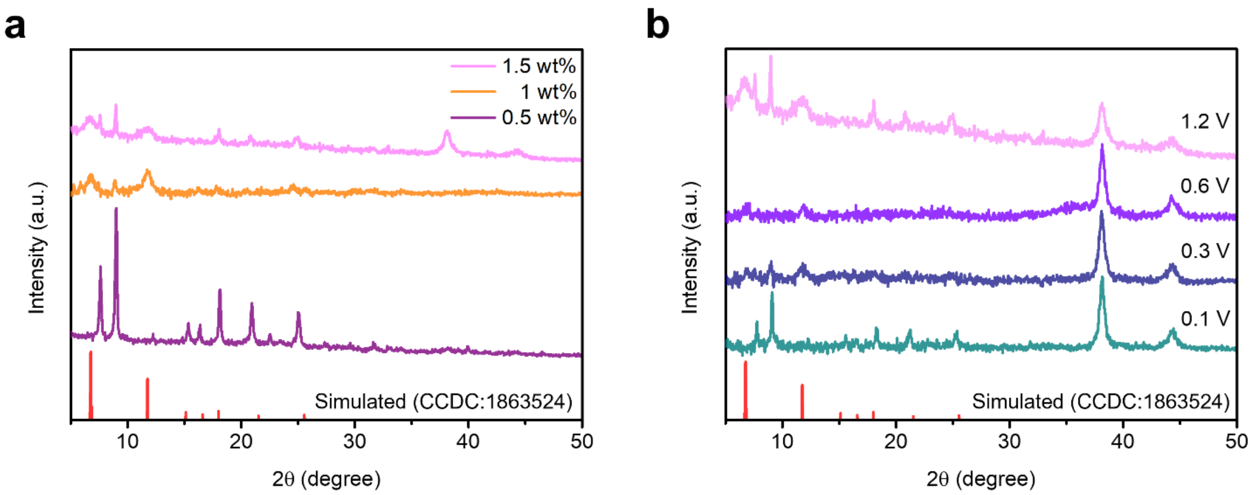

Supplementary Fig. 3 | Effect of alloy composition and voltage on the crystallinity of ZnMg-MOF-74.

a XRD patterns of ZnMg-MOF-74 synthesized using Mg-Ga alloys with different Mg contents.
b XRD patterns of ZnMg-MOF-74 synthesized from 1.5 wt% Mg-Ga alloy under different applied voltages.

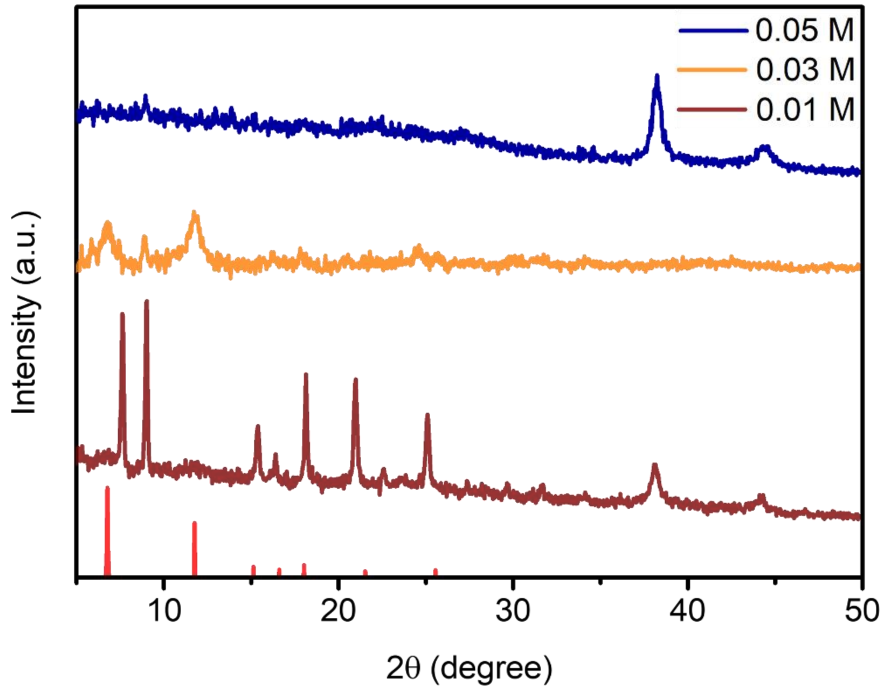

Supplementary Fig. 4 | Effect of ligand concentration on the crystallinity of ZnMg-MOF-74.

XRD patterns of ZnMg-MOF-74 synthesized with varying concentrations of H_4DOBDC ligand.

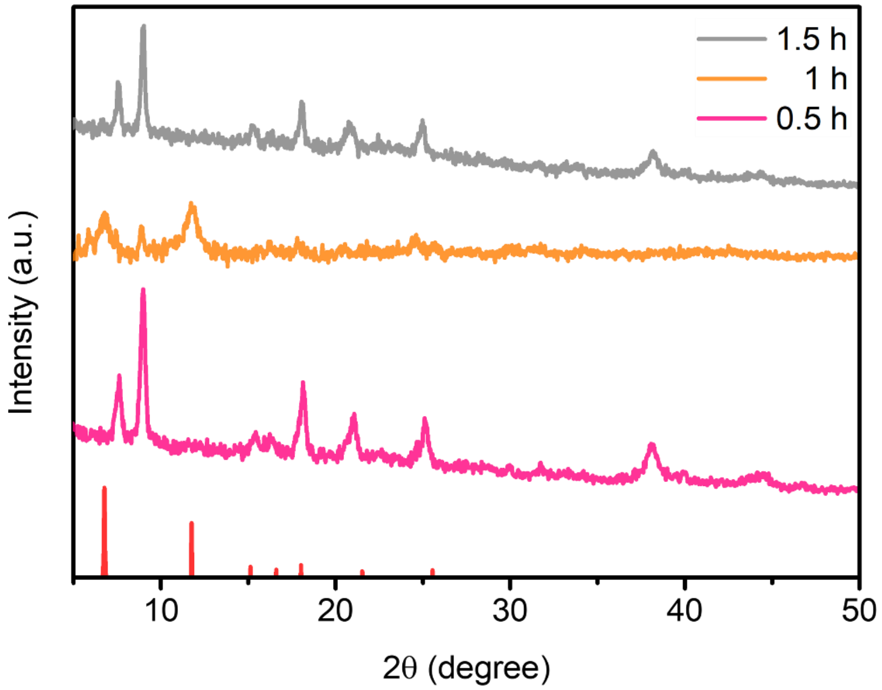

Supplementary Fig. 5 | Time-dependent crystallization of ZnMg-MOF-74.

XRD patterns of ZnMg-MOF-74 synthesized under different reaction durations.

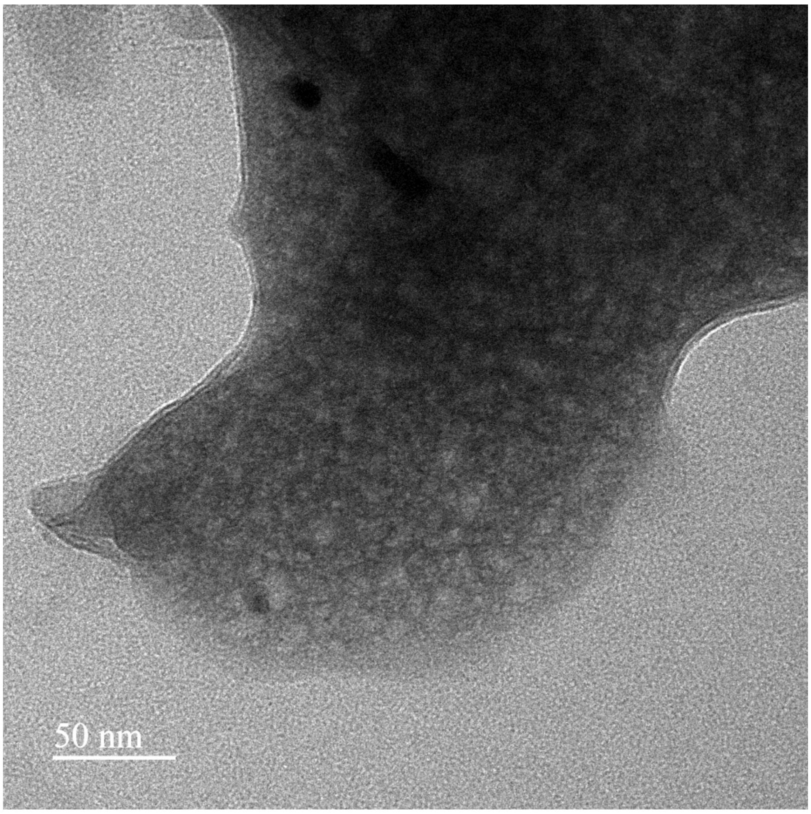

Supplementary Fig. 6 | Amorphous morphology of ZnMg-MOF-74 after direct annealing in air at 500 ℃.

TEM image showing the disordered, non-crystalline structure of ZnMg-MOF-74 after one-step thermal treatment under air atmosphere.

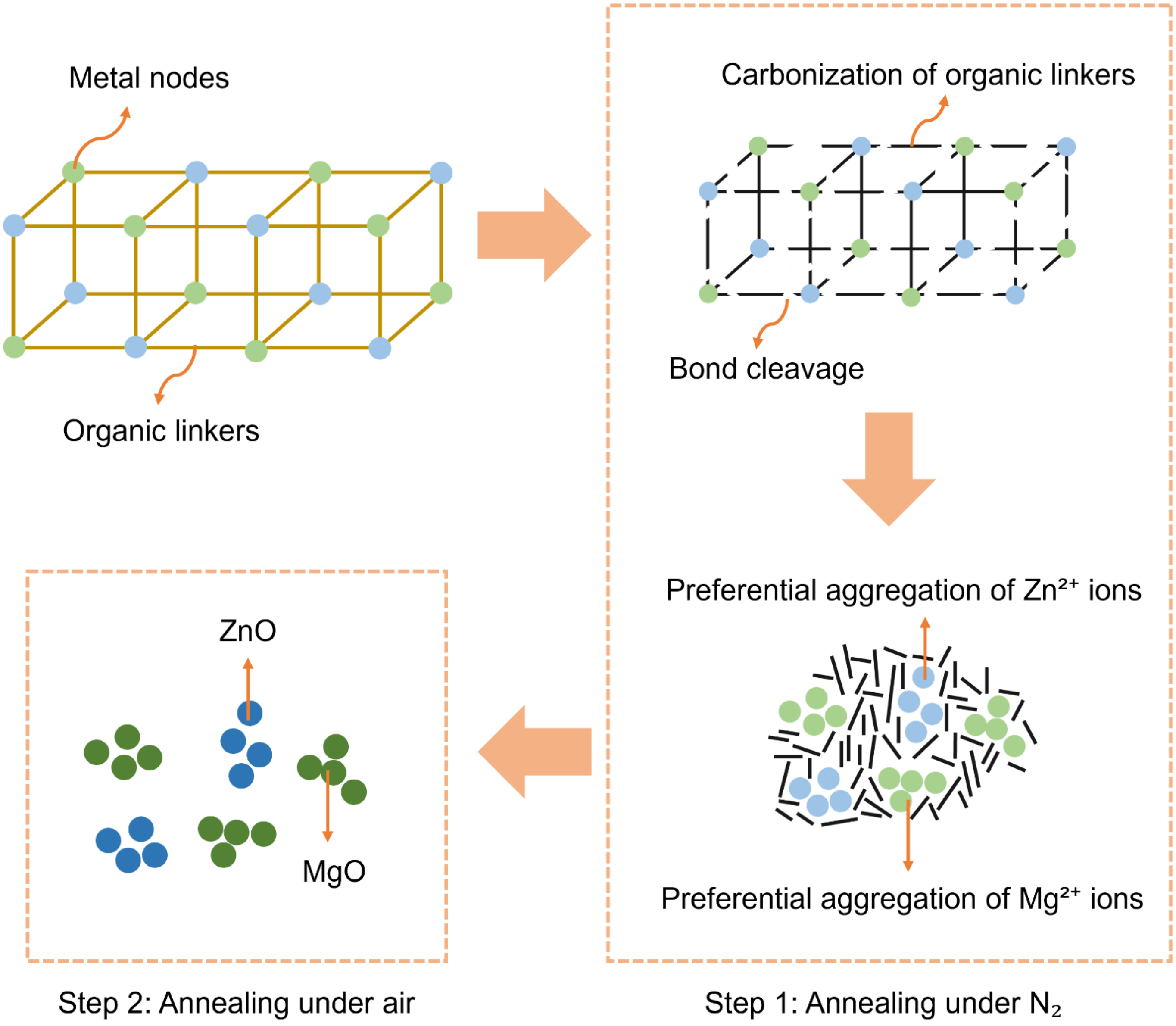

Supplementary Fig. 7 | Schematic illustration of the two-step annealing process converting ZnMg-MOF-74 into ZnMgO derivatives.

Step 1: Under N_2, organic linkers undergo carbonization and bond cleavage, leading to preferential aggregation of Zn^{2+} and Mg^{2+} ions within a partially carbonized matrix.

Step 2: Subsequent annealing in air removes the carbon matrix and yields separated ZnO and MgO crystalline domains.

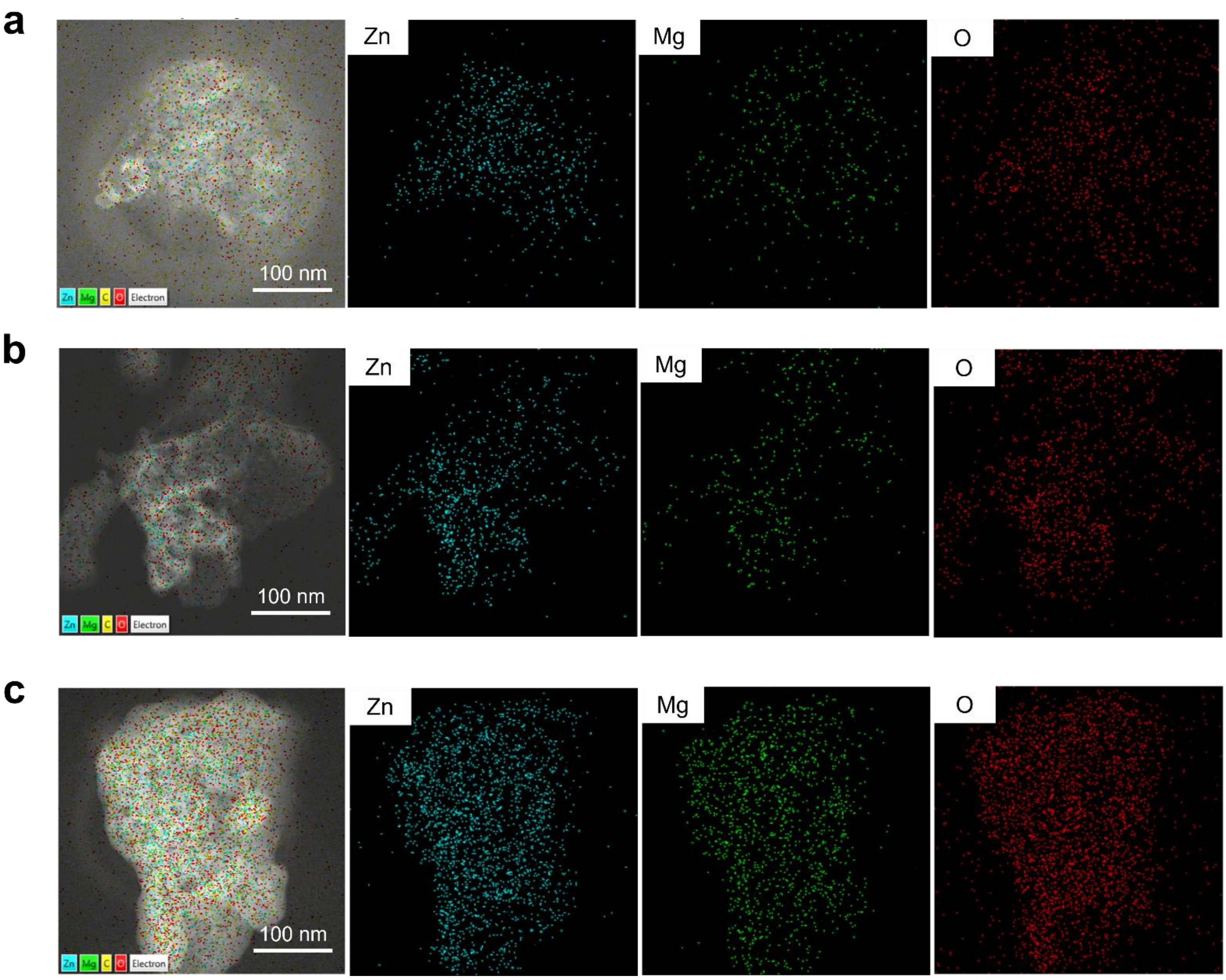

Supplementary Fig. 8 | TEM-EDS elemental mapping of ZnMgO derived from ZnMg-MOF-74 under different two-step annealing conditions.

a, ZnMg-350 N_2-500 Air; **b**, ZnMg-450 N_2-500 Air; **c**, ZnMg-550 N_2-500 Air. Zn (blue), Mg (green), and O (red) are uniformly distributed across particles, consistent with overall compositional homogeneity.

Supplementary Table 1 | Summary of synthesis conditions and applications of bimetallic MOFs.

Sample	Method	Condition	Major Findings	Ref.
CoM-MOF (M =Ni, Mn, Mg and Cu)	Ion exchange	200 ℃, 14 h	Cathode for aqueous Co-Zn batteries, delivering 344 mA h g^{-1} at 3 A g^{-1} and retaining 90% capacity after 1500 cycles at 20 A g^{-1}.	[1]
Co–Ni-MOF	One-pot synthesis	120 ℃, 24 h	Lithium-ion battery anode with Co/Ni = 1:1, delivering 800 mA h g^{-1} and retaining 98% capacity after 100 cycles at 100 mA g^{-1}.	[2]
Zn/Mg-MOF-74	One-pot synthesis	125 ℃, 30 h	CO_2 adsorption catalyst showing 128.3 cm^3 g^{-1} uptake at 273 K and >99% yield and selectivity for cyclic carbonate formation under 60 ℃, 0.8 MPa.	[3]
MnZn-MOF-74	One-pot synthesis	120 ℃, 24 h	Catalyst for direct oxidative carboxylation of styrene, achieving 92.0% conversion and 59.8% selectivity to styrene carbonate with high Mn^{3+} ratio (61.3%) under solvent-free conditions (90 ℃, 1.0 MPa CO_2, 4 h).	[4]
CoMn-MOF/NF	Electrodepositio n	-1.0 V, 400 s	Positive electrode for asymmetric supercapacitors, delivering 1091 C g^{-1} at 1 A g^{-1} and achieving an energy density of 84 Wh kg^{-1}.	[5]
NiS@LDH/NF	Electrodepositio n	-1.0 V	Core–shell electrocatalyst for overall seawater splitting, achieving 100 mA cm^{-2} at 1.636 V with nearly 100% Faradaic efficiency.	[6]
$Ni_{2.1}Cu_{0.9}(HITP)_2$	Electrodepositio n	-1 mA/cm^2, 24 h	Cathode for aqueous zinc–air batteries, delivering 706.2 mAh g^{-1} specific capacity and stable operation over 1250 cycles.	[7]
NiCo-MOF	Microwave-assisted synthesis	120 ℃, 850W	Bimetallic NiCo-MOF catalyst for oxygen evolution reaction, achieving overpotentials of 301 mV at 10 mA cm^{-2} and 328 mV at 300 mA cm^{-2} with excellent long-term stability.	[8]
ZnMg-MOF-74	Electrodepositio n	0.3 V, 1 h	The resulting MOF-derived ZnMgO material demonstrated enhanced photodetection with a high responsivity of 1.48 A/W and a fast rise time of 0.32 s.	This work

Supplementary Table 2 | Average grain area of ZnMgO under different pretreatment temperatures.

Average grain sizes were calculated from HR-TEM images after two-step annealing: nitrogen pretreatment at 350 ℃, 450 ℃, or 550 ℃, followed by air annealing at 500 ℃.

Sample	Average grain size (nm^2)
ZnMg-350N_2-500Air	7.17
ZnMg-450N_2-500Air	27.88
ZnMg-550N_2-500Air	3.66

Supplementary Table 3 | Photodetection performance of ZnMgO materials, including responsivity, external quantum efficiency, and detectivity.

Sample	Average Bright/dark current (nA)	Responsivity, R (A/W)	External quantum efficiency,EQE (%)	Detectivity, D*(Jones)
ZnMg-350N_2-500Air	-1.09×10−2/-2.56×10−3	7.64×10^{-4}	2.60×10^{-5}	2.60×10^{-5}
ZnMg-450N_2-500Air	$-2.16\times10^{3}/-5.47\times10^{2}$	1.48	5.04	4.08×10^{9}
ZnMg-550N_2-500Air	$-5.74\times10^{-3}/1.07\times10^{-5}$	5.26×10^{-6}	1.79×10^{-5}	1.03×10^{8}

1. Wang, H., Bai, J., He, Q., Liao, Y., Wang, S. & Chen, L. Crystal engineering of bimetallic cobalt-based metal–organic framework nanosheets for high-performance aqueous rechargeable cobalt–zinc batteries. *Journal of Colloid and Interface Science* **665**, 172-180 (2024).
2. He, S., Li, Z. & Wang, J. Bimetallic MOFs with tunable morphology: Synthesis and enhanced lithium storage properties. *Journal of Solid State Chemistry* **307**, 122726 (2022).
3. Gao, Z., Liang, L., Zhang, X., Xu, P. & Sun, J. Facile one-pot synthesis of Zn/Mg-MOF-74 with unsaturated coordination metal centers for efficient CO_2 adsorption and conversion to cyclic carbonates. *ACS Applied Materials & Interfaces* **13**, 61334-61345 (2021).
4. Gao, Z., Wang, H., Hu, Y. & Sun, J. Bimetallic MnZn-MOF-74 with enhanced percentage of Mn(III): Efficient catalytic activity for direct oxidative carboxylation of olefins to cyclic carbonates under mild and solvent-free condition. *Journal of Colloid and Interface Science* **671**, 232-247 (2024).
5. Moradi, M., Afkhami, A., Madrakian, T. & Moazami, H. R. Electrosynthesis of CoMn layered-double-hydroxide as a precursor for Co-Mn-MOFs and subsequent electrochemical sulfurization for supercapacitor application. *Journal of Energy Storage* **71**, 108177 (2023).
6. Ren, J. T. et al. Rational synthesis of core-shell-structured nickel sulfide-based nanostructures for efficient seawater electrolysis. *Small* **19**, 2300194 (2023).
7. Liu, M. et al. Electrodeposition of Ni/Cu bimetallic conductive metal–organic frameworks electrocatalysts with boosted oxygen reduction activity for zinc-air batteries. Small 20, 2405309 (2024).
8. Beglau, T. H. Y., Fei, Y. & Janiak, C. Microwave-assisted ultrafast synthesis of bimetallic nickel-cobalt metal–organic frameworks for application in the oxygen evolution reaction. *Chemistry - A European Journal* **30**, e202401644 (2024).
